# Evaluation of the Antifungal and Biochemical Activities of Fungicides and Biological Agents against Ginseng *Sclerotinia* Root Rot Caused by *Sclerotinia nivalis*

**DOI:** 10.3390/microorganisms12091761

**Published:** 2024-08-25

**Authors:** Shi Feng, Chunlin Wang, Zhaoyang Xu, Baozhu Dou, Xue Wang, Lina Yang, Baohui Lu, Jie Gao

**Affiliations:** 1 College of Plant Protection, Jilin Agricultural University, Changchun 130118, China; 20211671@mails.jlau.edu.cn (S.F.); doubaozhu0402@163.com (B.D.); xuew@jlau.edu.cn (X.W.); yanglina20140@126.com (L.Y.); 2 State-Local Joint Engineering Research Center of Ginseng Breeding and Application, Changchun 130118, China

**Keywords:** *Sclerotinia nivalis*, fungicides, inhibitory activity, control effects, biochemical mechanisms, *Panax ginseng*

## Abstract

The objective of this study was to identify effective agents for the prevention and control of ginseng *Sclerotinia* root rot disease caused by *Sclerotinia nivalis*. The inhibitory effects of 16 chemical fungicides and 10 biocontrol agents (strains) on mycelial growth and sclerotium formation in *S. nivalis* were determined using a plate confrontation essay. The results showed that the best chemical agents for inhibiting the mycelial growth and sclerotium formation of *S. nivalis* were fluconazole and fludioxonil, while *Bacillus amyloliquefaciens* FS6 and *B. subtilis* (Kono) were the best biocontrol agents (strains). The results of field trials in 2022 and 2023 showed that the control effects of fluconazole and fludioxonil on ginseng *Sclerotinia* root rot disease were 90.60–98.16%, and those of the biocontrol agents *B. amyloliquefaciens* FS6 and *B. subtilis* (Kono) were 94.80–97.24%, respectively. Chemical agents produced abnormal and twisted mycelia, while the biocontrol agents increased mycelial branching, dilated the mycelium tip, and revealed an abnormal balloon. All of the fungicides decreased the ergosterol content, changed the cell membrane permeability, and increased the protein and nucleic acid permeability. These results suggest that these are potential agents for controlling ginseng *Sclerotinia* root rot disease, and their biochemical mechanisms of chemical and biocontrol of this disease were demonstrated.

## 1. Introduction

Ginseng (*Panax ginseng* C. A. Meyer) is a perennial herb of Acanthopanax senticosus, and it is known as “the king of all herbs”. The saponins and polysaccharides in ginseng can regulate the central nervous system, improve learning and memory abilities, help resist fatigue, and enhance the body’s immunity. Polyacetylene compounds and diol saponins can resist oxidation. The enol, alkynetriol, and volatile oil substances in ginseng can play a role in its antitumor effects [1,2]. Ginseng is mainly distributed in China, Russia, and South Korea, where it is an important part of the local diversified farming economy [3].

Ginseng is mainly distributed in Jilin, Liaoning, and Heilongjiang Provinces in China, with Jilin Province accounting for 40% of the total planting area. It is a genuine medicinal material in the northeastern region. Currently, the total area of artificial cultivation of Chinese ginseng covers more than 133 thousand hectares, with an output accounting for more than 80% of the world’s total output [4]. Ginseng has a long growth cycle and is susceptible to various diseases, including ginseng *Sclerotinia* root rot, which is one of the most significant soil-borne root diseases [5].

*Sclerotinia* is a worldwide necrotrophic pathogenic fungus that infects more than 600 kinds of crops every year and causes serious losses to agricultural production worldwide [6,7]. *Sclerotinia nivalis* infected ginseng in Korea in 2013, leading to *Sclerotinia* root rot [8], and it was reported to infect American ginseng in China in 2021 [9]. Due to the lack of disease-resistant varieties, various fungicides with different mechanisms of action, such as dicarboxyimide (iprodione), have been utilized. Sterol biosynthesis inhibitors (hexaconazole and prochloraz), succinate dehydrogenase inhibitors (boscalid), methoxyacrylates (trifloxystrobin), and triazoles (propiconazole and flusilazole) have been used to control *Sclerotinia* diseases in different crops [10,11,12,13,14,15,16]. However, few reports are available on the effective control of ginseng *Sclerotinia* root rot disease. Furthermore, long-term use and excessive application of fungicides accelerate resistance to them, resulting in the decline or failure of control. Therefore, alternative chemicals are needed for the successful and sustainable management of *Sclerotinia* diseases. Biological agents have been commonly used to control *Sclerotinia* diseases in recent years, such as *Trichoderma harzianum*, *Bacillus amyloliquefaciens*, *B. subtilis*, and *B. methylotrophicus* [17,18,19,20]. Biological agents have become a research hotspot in disease prevention and treatment due to their advantages of leaving no pesticide residue, causing no drug resistance, and having environmental friendliness.

Chemical fungicides were selected in this study based on the mechanisms of action of different agents, and for the first time, the interference of factors such as additives in commercial agents was eliminated in the experiments. In addition, commercial biological control agents, as well as biological control strains isolated from the rhizosphere soil of ginseng and tobacco that were confirmed to have broad-spectrum antagonistic effects in our laboratory, were also selected in this study for indoor tests of the antifungal effects and for field efficacy trials in order to screen highly effective chemical agents and biological control strains for the prevention and control of ginseng *Sclerotinia* root rot, thus providing a reference for field control and pesticide registration.

## 2. Materials and Methods

### 2.1. Materials

The pathogenic fungus used in this study was *Sclerotinia nivalis* YC5 from the Phytopathology Laboratory of Jilin Agricultural University. The samples were isolated from diseased *P. ginseng*. Sixteen kinds of chemical fungicides and 10 kinds of biological agents (strains) were tested. The manufacturers are shown in Table 1 and Table 2.

### 2.2. Determination of the Indoor Toxicity of the Chemicals

After dissolving the chemicals in 0.1% acetone, they were diluted with 0.1% Tween 80 (Beijing Solarbio Technology Co., Ltd., Beijing, China) into solutions with concentrations of 10^4^, 10^3^, 10^2^, 10^1^, 10^0^, and 10^−1^ mg/L. The prepared 1.0 mL solutions were added to 9.0 mL of potato dextrose agar (PDA) medium, shaken, and poured into a Petri dish to make a drug-containing plate [21,22]. Instead, an equal amount of sterile water was added to the control group. The concentration of the re-screening agent was determined according to the initial screening results, as shown in Table 3.

The colony diameters of *Sclerotinia* spp. treated with different concentrations of chemical agents were obtained by using the mycelial growth rate method, and the respective inhibition rate was calculated based on the colony diameter of the control.

The inhibitory rate (%) = 100 × (colony diameter of control group − colony diameter of treatment group)/colony diameter of control group.

Then, three variables were established in the SPSS 26.0 software—concentration, inhibition rate, and total number—and the corresponding data were entered into the respective variables. On the “probit analysis” page, the inhibition rate was selected as the response frequency, the total number was selected as the observation value, concentration was selected as the covariate, the base of 10 logarithms was selected as the conversion choice, and the logit was selected as the model choice. Finally, OK was clicked to calculate the EC_50_.

### 2.3. Determination of the Indoor Toxicity of the Biocontrol Agents

The commercial agents were directly diluted with sterile water to six concentrations of 10^6^, 5 × 10^5^, 10^5^, 5 × 10^4^, 10^4^, and 10^3^ CFU/mL depending on the number of live bacteria; the biocontrol strains isolated in the laboratory (Table 1) were cultured in LB medium at 28 °C and 180 r/min for 12 h to obtain a seed liquid. A fermentation broth was obtained by inoculating 4.0% of the inoculum in prepared LB medium and culturing at 28 °C and 180 r/min for 16 h. Then, 1.0 mL of the prepared concentration was added to 9.0 mL of heated PDA medium, which cooled to about 40 °C. The solutions were shaken well and poured into Petri dishes to make plates containing live bacteria [23]; instead, an equal amount of sterile water was added to the control group. The determination of the inhibition rate and the method of calculating EC_50_ and EC_90_ were the same as those described in Section 2.2.

### 2.4. Effects of the Different Treatments on the Formation of Sclerotia by Sclerotinia nivalis

*S. nivalis* inoculated on fungicide-containing plates was cultured for 30 days, and the number of sclerotia formed was counted and compared with that of the control [24]. The inhibition rate was calculated, and the effects of the agents and the concentrations on the formation of sclerotia were analyzed.

### 2.5. Protective and Curative Efficacy In Vitro

Four-year-old ginseng roots were washed and disinfected with 75% alcohol and then washed three times in sterile water (sterile water was obtained by sterilizing distilled water at 121 °C for 1 h). The mycelia were inoculated in vitro.

Protective effect: The ginseng was disinfected and then sprayed at high, medium, or low concentrations. Tween 80 (0.1%) was used as a control. All ginseng roots were sprayed with 2 mL of *S. nivalis* YC5 24 h after spraying.

Curative effect: The disinfected ginseng roots were sprayed with 2 mL of the different concentrations of each fungicidal solution 24 h after inoculating with 1 mL of the *S. nivalis* YC5 mycelium solution. The chemical agent was sprayed with 0.1% Tween 80 as the control, and the biocontrol agent was sprayed with an LB culture medium [25,26]. Five ginseng roots were inoculated in each treatment, and each treatment was repeated three times. The incidence was observed, and the control effect was calculated on days 3, 7, and 10, respectively. The disease index was classified as follows:

0: Healthy, no visible lesions;

1: Healthy appearance, with only a small amount of white mycelium covering the surface of the ginseng roots;

3: 1–2 water-soaked lesions with diameters of <2 cm, and the internal tissue around the affected site beginning to soften;

5: More than two water-soaked lesions or more than a single lesion that was >2 cm in diameter, and the tissues around the affected site were soft and loose. The rotting area of the root was no more than 30%;

7: 30–50% of the rotting area was soft, and the white mycelium on the surface began to assume a spherical shape;

9: Soft rotted area of more than 50%, a large number of mycelia, and black sclerotia that formed on the root surface.

The disease index and control efficacy were calculated using Formulas (1) and (2).

Disease index = 100 × ∑(number of diseased roots at all levels × the disease grade)/ (total root number × maximum grade)(1)

Control efficacy (%) = 100 × (disease index in control − disease index in treatment)/ disease index in control(2)

### 2.6. Control Efficacy of the Agents on Ginseng Sclerotinia Root Rot in a Field Trial

Chemicals with obvious effects on EC_90_ and biocontrol agents were selected to protect the roots at 10 times the EC_90_ concentration. The experiment was carried out at the ginseng experimental field of Jilin Agricultural University (125°410385′ E, 43°810433′ N) on 4-year-old ginseng (cv: Damaya). Each agent was replicated in three plots, each plot area was 1.5 m^2^, and the plots were randomized. All ginseng roots were injected with 300 mL and 24 h after administration, and the roots were inoculated with the *S. nivalis* mycelium suspension (0.1 g/mL). After 14 days of irrigation, all ginseng roots in each plot were dug out to calculate the incidence, and the control effect was determined [27,28]. The methods of calculating the disease index and control efficacy were the same as those in Section 2.5.

### 2.7. Biochemical Action of the Agents on S. nivalis YC5

*Effects of the agents on the morphology of the mycelium*: The morphology of the mycelium when treated with the different agents was observed under a Zeiss microscope after culturing at 25 °C for 3 days. After the chemical/biological treatments, the mycelia were picked and put on a glass slide; 30–40 μL of sterile water was added, and then the slide was covered. The morphology of the mycelium was directly observed under the microscope without staining. The samples were magnified 400 times and observed. Three repeats were employed for each process.

*Effects of the agents on mycelial membrane permeability*: Preparation of the mycelial sample: First, 1 mL of the mycelium suspension (25 °C and 150 r·min^−1^ for 5 days) was inoculated in PDB medium after sterilization and cultured in a constant-temperature rotary shaker at 25 °C and 150 r·min^−1^ for 48 h. Then, the culture was harvested and centrifuged at 8000 r·min^−1^ at 4 °C for 5 min. The supernatant was discarded, the mycelia were washed three times with sterile water to remove the medium, and then they were collected again through centrifugation.

The relative conductivity of the mycelia was determined in a culture medium according to the method of Mao et al. [29]. Two grams (fresh weight) of mycelia were suspended in 20 mL of PBS buffer (50 mmol·L^−1^ pH 7.2), and different concentrations of the chemicals were added. Nothing was added to the control group. Each concentration was repeated three times. Under the conditions of 25 °C and 150 r·min^−1^, the electrical conductivity was measured after sampling every 2 h. After 24 h, the mycelial suspension was boiled for 10 min to inactivate it, and the conductivity was recorded after cooling. The relative conductivity was measured each time and was used to express cell membrane permeability. The formula for calculating the relative conductivity was as follows:

Relative conductivity (%) = [(Lt − L0) (Le − L0)] × 100

where Lt represents the electrical conductivity of the culture medium at a particular time, L0 represents the electrical conductivity measured at 0 h, and Le represents the electrical conductivity of the bacterial suspension after boiling and cooling.

*Leakage of nucleic acid and protein from hyphae after treatment with different agents*: The leakage of protein and nucleic acids into the culture medium was detected according to Zhang’s method [30]. Two grams of mycelia (fresh weight) were suspended in 20 mL of PBS buffer (50 mm, pH 7.2), and different concentrations of chemicals were added. The non-fungicide group was used as the blank control group, and three parallel samples were set for each concentration. After 24 h of incubation at 25 °C and 150 r·min^−1^, the bacterial suspension was centrifuged at 8000 r·min^−1^ and 4 °C for 10 min. The absorbance of the supernatant was measured at 260 nm and 280 nm with a spectrophotometer.

*Effects of different agents on ergosterol*: Test treatment: The hyphae of *Sclerotinia nivalis* YC5 cultured on a solid medium for 5 days were shaken at 25 °C in PDB medium for 7 days. The wet hyphae were dried with absorbent paper and weighed as 1 g of hyphae. Then, they were treated with chemicals at different concentrations, prepared in sterile water, and cultured for 4 days; then, they were dried, ground, and crushed, and 0.1 g was weighed out for later use.

Sample treatment: The ratio of material to liquid was 1:50 (1 g of mycelia were added to 50 mL of methanol). The sample was soaked for 2 h before ultrasound at a power of 200 W for 10 min. Then, the crude sample was filtered with a 0.22 μm organic membrane [31].

Chromatographic conditions: C18 column, injection temperature of 35 °C, injection volume of 5 μL, mobile phase A: 0.1% acetic acid in water, mobile phase B: methanol; flow rate: 0.8 mL; detection wavelength: 282 nm. The ergosterol content in the sample was determined.

### 2.8. Data Analysis

All data were analyzed with the SPSS software (SPSS Inc., Chicago, IL, USA). The EC_50_ and EC_90_ values were calculated using the inhibitory rate of the log_10_ concentration of fungicide in a regression analysis. Analysis of variance and Fisher’s minimum significant difference (LSD) test were used to detect differences. A *p*-value of <0.05 was considered significant.

## 3. Results

### 3.1. Indoor Sensitivity of the Chemical Fungicides

The mass concentrations of the 16 fungicides shown in Table 2 were used. Among them, thiram was used as the control agent. When the colony growth diameter was measured, the blank control had already filled the dish, which had a diameter of 90 mm. The results indicated that the 16 chemical fungicides inhibited the growth of *Sclerotinia nivalis* YC5 mycelium (Table 4). The lowest EC_50_ values (<0.1 mg/L) were assigned to epoxiconazole, fluazinam, dimethachlon, fludioxonil, difenoconazole, fluxapyroxad, tebuconazole, and myclobutanil. The agents with the lowest EC_90_ values (<1.0 mg/L) were epoxiconazole, fluazinam, dimethachlon, fludioxonil, difenoconazole, fluxapyroxad, and tebuconazole. Therefore, these data can be used in the primary considerations of the comprehensive control of *S. nivalis*. Epoxiconazole and fluazinam can be used as the first choice for field experiments because of their small and stable EC_50_ and EC_90_ values. Thiram and trifloxystrobin had a minimal effect on mycelial growth and may have little effect on the control of *S. nivalis* in actual production.

### 3.2. Screening Results of the Biocontrol Agents (Strains)

The results of the indoor tests showed that there were large differences in the inhibitory effects of the different biocontrol agents (strains) on the mycelial growth of *S. nivalis* YC5 (Table 5). *Trichoderma harzianum* T-22 was used as a control agent. When the colony growth diameter was measured, the blank control had already filled the dish, which had a diameter of 90 mm. The EC_50_ values of the other biocontrol agents (strains) were smaller, except for starch *Bacillus amyloliquefaciens* B7900 and *Trichoderma harzianum* (T-22). This may have been related to the longer time it took for the biocontrol bacteria to complete the adaptation and colonization process and exert an optimal biocontrol effect. The specific reasons need to be further analyzed. Based on the results of the EC_50_ and EC_90_ values, it was recommended to select *Bacillus subtilis* (Kono) and *B. amyloliquefaciens* FS6 for the field efficacy trials.

### 3.3. Effects of Different Fungicides on the Number of Sclerotia

The fungicides had different effects on the formation of sclerotia, and the number of sclerotia decreased with the increase in the drug concentration. The number of sclerotia formed by *S. nivalis* YC5 in the chemical agent fludioxonil was the lowest; the average inhibition rate of the formation of sclerotia was 89.74%. This was followed by fluxapyroxad, which had an average inhibition rate of 88.81% (Table 6).

Overall, the effect of the biocontrol agents on the formation of sclerotia was greater than that of the chemicals (Table 7). *S. nivalis* YC5 did not form sclerotia after 30 days in compound endophytic *Paenibacillus polymyxa* (Linyi), *B. subtilis* (Kono), *B. subtilis* (De qiang), and *B. amyloliquefaciens* FS6. The rate of inhibition of sclerotium formation was 100.00%. The number of sclerotia formed in *P. polymyxa* KN-03, *B. subtilis* Y4, and *B. amyloliquefaciens* B7900 was also relatively low. The average number of sclerotia formed in *P. polymyxa* KN-03 and *B. subtilis* Y4 was lower than the average number that formed in fludioxonil, with inhibition rate of 95.90% and 97.20%, respectively.

### 3.4. Protective and Curative Efficacy

**Figure 1:**
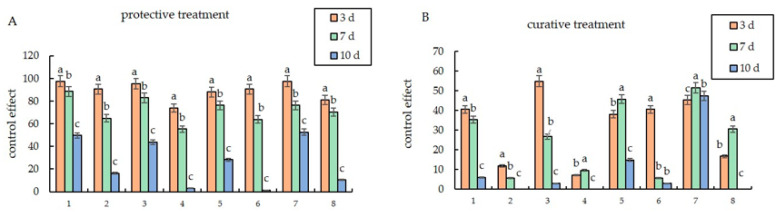
In vitro inoculation to determine the control effects of different agents on *Sclerotinia nivalis* YC5: (**A**) protective effect; (**B**) curative effect. Note: In the abscissa, 1 represents epoxiconazole with the concentration of 0.052 mg/L (high concentration), 2 represents epoxiconazole with the concentration of 0.0052 mg/L (low concentration), 3 represents fludioxonil with the concentration of 5.353 mg/L, 4 represents fludioxonil with the concentration of 0.5353 mg/L, 5 represents *Bacillus amyloliquefaciens* FS6 with the concentration of 3.5505 × 10^9^ CFU/mL, 6 represents *B. amyloliquefaciens* FS6 with the concentration of 3.5505 × 10^8^ CFU/mL, 7 r epresents *B. subtilis* (Kono) with the concentration of 2.0676 × 10^9^ CFU/mL, and 8 represents *B. subtilis* (Kono) with the concentration of 2.0676 × 10^8^ CFU/mL. The letters a, b, c represent different levels of differences between treatments. The treatment with the highest average effectiveness over different time periods is labeled as “a”. Then, each average is compared with this reference average. If the difference is not significant, it is also labeled as “a”. This process continues until one average is significantly different, which is labeled as “b”. The rest of the averages are labeled accordingly in a similar manner.

The results of the tests on the chemical and biocontrol agents showed that the control effect increased with the increase in the treatment concentration (Figure 1). At the same concentration, the protective effects of different agents on *S. nivalis* YC5 were greater than the curative effects. On day 3, the protection rate approached 100%, which was significantly higher than the cure rate, which remained low. On day 7, the rate of protection by epoxiconazole and fludioxonil at high concentrations against *S. nivalis* YC5 was more than 80%, while the cure rate of epoxiconazole was only about 30%. The cure rate of fludioxonil was more than 25%, while the protection rate of the biocontrol agents at high concentrations was more than 67%, and the curative effect was more than 45%, which was 44.4% higher than that of the chemical treatment. The protective effect was better than the curative effect as a whole, and the protective and curative effects gradually decreased over time; the control effect experienced a sharp decline after 10 days of chemical treatment.

### 3.5. Effects of Different Agents on the Field Control of Sclerotia nivalis

**Figure 2:**
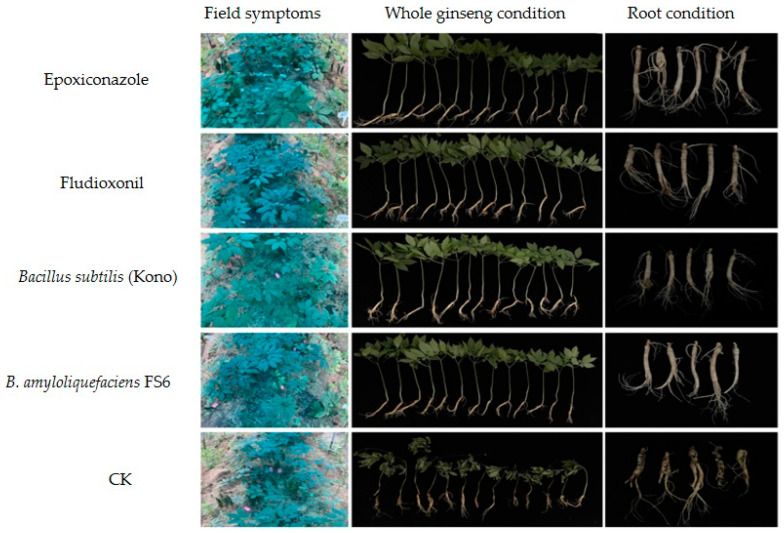
Control effects of the different fungicides on *Sclerotinia nivalis* YC5 in the field (Changchun, June 2022). All ginseng roots were injected with 300 mL, and 24 h after administration, the roots were inoculated with the *S. nivalis* mycelium suspension (0.1 g/mL). Each agent was replicated in three plots, and the plots were randomized.

**Figure 3:**
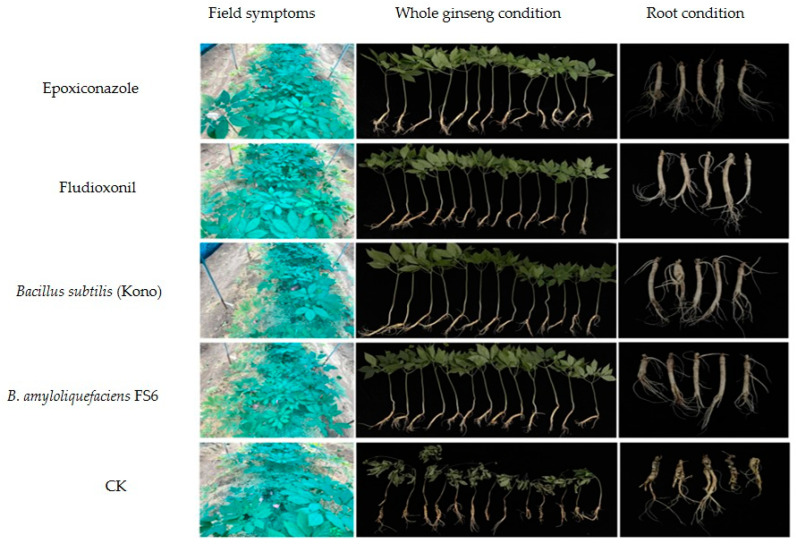
Control effects of the different fungicides on *Sclerotinia nivalis* YC5 in the field (Changchun, August 2023). All ginseng roots were injected with 300 mL, and 24 h after administration, the roots were inoculated with the *S. nivalis* mycelium suspension (0.1 g/mL). Each agent was replicated in three plots, and the plots were randomized.

After 24 h of administration, the roots were inoculated with the *S. nivalis* YC5 mycelial suspension (0.1 g/mL), and all ginseng plants were dug out 14 days after 50 mL of irrigation. The results of the two field trials showed that the field control effect of the chemical agents was 90.60–98.16%, while the field control effect of the biocontrol agents was 94.80–97.24%. The four agents had excellent control effects on ginseng *Sclerotinia* root rot disease caused by *S. nivalis* (Table 8, Figure 2 and Figure 3).

### 3.6. Effects of the Different Agents on Mycelial Morphology

**Figure 4:**
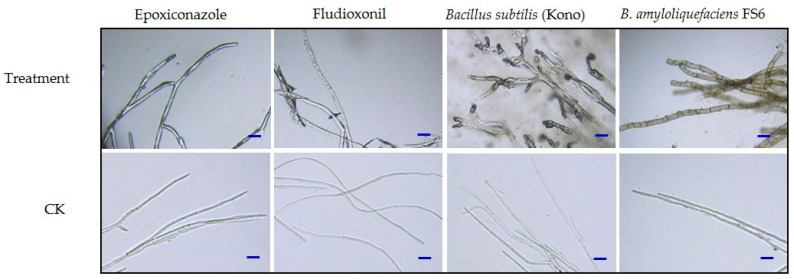
Influence of agents on the mycelial morphology of *Sclerotinia nivalis* YC5. The increased subbranches of the mycelia are marked with red arrows. The images were obtained with a microscope. Bar = 20 μm.

After treatment with the chemical agents, the hyphae of *S. nivalis* YC5 were twisted and shriveled, whereas the hyphae treated with the biological agents became thicker, their color deepened, balloon-like bubbles appeared at their tips, and there were some other changes, such as the outflow of inclusions. In contrast, the control mycelia were uniformly thick and slender, and they had a uniform distribution of contents (Figure 4).

### 3.7. Effects of Different Agents on the Mycelial Membrane Permeability of Sclerotinia nivalis

**Figure 5:**
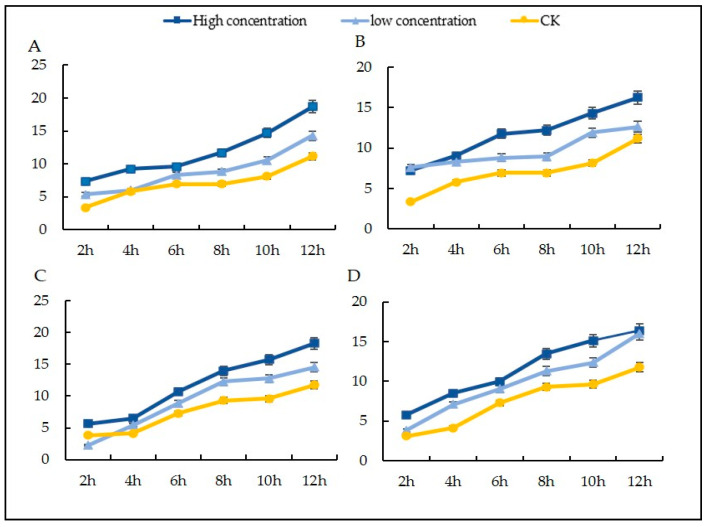
Effects of the fungicides on mycelial membrane permeability: (**A**) epoxiconazole; (**B**) fludioxonil; (**C**) *Bacillus subtilis* (Kono); (**D**) *B. amyloliquefaciens* FS6.

The effects of the chemical and biological agents on the permeability of the cell membranes were investigated by detecting the conductivity of the mycelia. The conductivity of *S. nivalis* increased with the increase in treatment concentration and time (Figure 5). This result suggested that those agents damaged the cell membrane, leading to the leakage of electrolytes within the mycelial cells.

### 3.8. Leakage of Nucleic Acids and Proteins in Mycelia after Treatment with Different Agents

**Figure 6:**
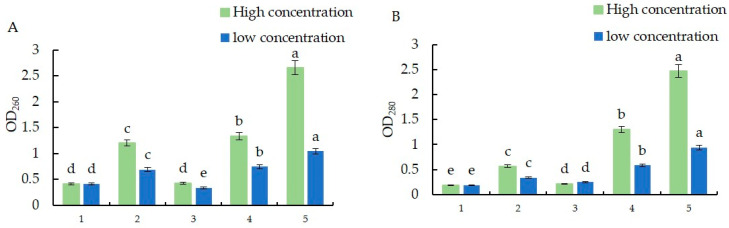
(**A**) Nucleic acid leakage of *Sclerotinia nivalis* YC5 when treated with different agents; (**B**) protein leakage of *S. nivalis* YC5 when treated with different agents. Note: In the abscissa, 1 represents the control, 2 represents the fungicide epoxiconazole, 3 represents the fungicide fludioxonil, 4 represents the *Bacillus amyloliquefaciens* FS6, and 5 represents the *B. subtilis* (Kono). The letters a–e represent different levels of differences between treatments. The treatment with the highest average effectiveness over different time periods is labeled as “a”. Then, each average is compared with this reference average. If the difference is not significant, it is also labeled as “a”. This process continues until one average is significantly different, which is labeled as “b”. The rest of the averages are labeled accordingly in a similar manner.

The nucleic acid leakage when using the different agents was greater than that in the control group, and the *S. nivalis* YC5 nucleic acid leakage of *B. subtilis* (Kono) treated with 10^7^ CFU·mL^−1^ was 6.3 times greater than that of the control, followed by *Bacillus amyloliquefaciens* FS6. The protein leakage trend was consistent with that of nucleic acid leakage, indicating that the drug treatments increased the cell membrane permeability of the mycelia of *S. nivalis*, resulting in the leakage of nucleic acids and proteins through the cell membrane. The leakage of nucleic acids due to the fludioxonil treatment was similar to that of the CK treatment. Fludioxonil may have acted on nucleic acids and proteins to oxidize and dissolve them, so they were not detected. Unlike the results for the biocontrol bacteria, the results for the chemical fungicides were consistent with the trend of changes in electrical conductivity (Figure 6).

### 3.9. Effects of the Different Agents on Ergosterol

**Figure 7:**
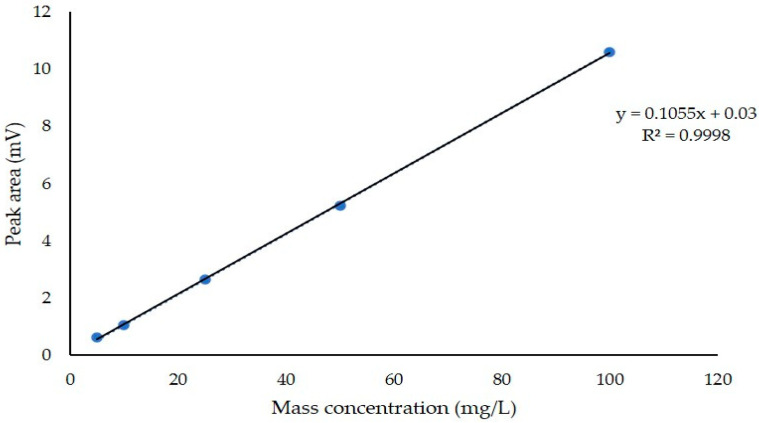
Establishment of the standard curve for ergosterol.

A standard solution of ergosterol was diluted and analyzed under the chromatographic conditions described above. The regression equation, correlation coefficient, and linear range of endogenous hormones were calculated. The peak area was the ordinate (y), and the mass concentration was the abscissa (x, mg/L). The regression equation was y = 0.1055x + 0.03, R^2^ = 0.9998. The results indicated a good correlation between the standard concentration and the peak area (Figure 7).

With the untreated strains as the control, the samples were injected under the chromatographic conditions described above, the peak area was recorded, and the ergosterol contents of the different concentrations were calculated.

Significant differences in ergosterol content were detected in the different treatments, in which the ergosterol content of the strains cultured with drugs was significantly lower than that of the strains cultured without fungicides (Table 9). The ergosterol content of the strains was positively correlated with the concentration after treatment with fludioxonil and epoxiconazole. The ergosterol content of the strain cultured with epoxiconazole was significantly lower than that of the strain cultured without a fungicide, and the ergosterol content was positively correlated with the concentration of the treatments with the four agents. The ergosterol content of the strain treated with a low concentration of epoxiconazole was 4.2 μg·mL^−1^; the content of ergosterol decreased significantly compared with the control (197.87 μg·mL^−1^).

## 4. Discussion

In this study, 16 chemical fungicides were selected, and the growth rate method was used to study their antifungal activity against *S*. *nivalis*. The results showed that 13 chemicals—epoxiconazole, trimethoprim, tebuconazole, fluidimidine, dimethachlon, fluazolamide, difenoconazole, azoxystrobin, pyrazole, thiophanate, thiophanate, benzoin, and methyl thiophanate—had inhibitory effects on mycelial growth in *S. nivalis*. The antifungal effects of epoxiconazole, fluazolamide, and thiram on *S. nivalis* had not been previously reported. Therefore, these fungicides could be used for subsequent field efficacy trials and were expected to serve as a reference for field applications. Wang reported that 10% difenoconazole WG and 70% methyl thiophanate had good antifungal effects on *Sclerotinia* disease in 2014 [32]. Wang found that fluxapyroxad had a strong inhibitory effect on *S. sclerotiorum* isolated from soybean, with values of the mean effective concentration for 50% inhibition (EC_50_) ranging from 0.021 to 0.095 µg/mL [33]. The distribution of the sensitivity of *Sclerotinia sclerotiorum* populations to fluazinam was determined using 103 strains collected from fields in Jiangsu Province of China in 2018. The average EC_50_ value of fluazinam against these strains was 0.0073 ± 0.0045 μg/mL for mycelial growth [29]. This study further confirmed the antifungal effects of these agents. The agents used in this study were all original fungicides, allowing the antifungal effects of various fungicides on ginseng *Sclerotinia* root rot disease to be more fully shown by eliminating the interference of some auxiliaries and additives. In addition, the inhibitory effects of 10 different biocontrol agents (strains) on the mycelial growth of *S. nivalis* YC5 were studied. The EC_90_ values of the biocontrol agents (strains) were greater, which may have been related to the time that the biocontrol bacteria took to complete adaptation and colonization and to exert the best biocontrol effect. The specific reasons need to be further analyzed.

The sclerotium is an important structure that is formed when *Sclerotinia* fungi resist an adverse environment; thus, it is not only a vegetative storage organ but also a dormant body for adverse environmental conditions. The sclerotia are hard and difficult to remove, so if the number of sclerotia formed can be reduced, a control effect can be achieved. In this study, biocontrol agents, including endophytic *Paenibacillus polymyxa* (Linyi), *B. subtilis* (Kono), and *B. amyloliquefaciens* FS6, completely inhibited the formation of sclerotia in *S. nivalis* YC5 over 30 days. Sclerotia are asexual structures in *Sclerotinia* fungi that enable them to survive over winter and accumulate, resist adversity, and survive in the soil for many years [34]. Treating soil with a biocontrol agent in the field can effectively reduce sclerotium formation to achieve the purpose of controlling ginseng *Sclerotinia* root rot.

Fungicides with protective and curative effects control the occurrence and spread of diseases more effectively [35]. The chemical agents epoxiconazole and fludioxonil, as well as starch spores (FS6) and *Bacillus subtilis* (Kono) as biocontrol agents, had satisfactory protective effects on *Sclerotinia nivalis*, demonstrating that these agents have the potential to be used to control *S. nivalis* in the field. The protective effect on *S. nivalis* YC5 was significantly greater than that of the other treatments at the same concentration. Therefore, to better control the disease, chemicals or biological agents should be irrigated in advance or during the early stage of the spread of ginseng *Sclerotinia* root rot disease. The results of two years of field trials also showed that the preventive effect of root irrigation with two chemical agents, epoxiconazole and fludioxonil, as well as two biological agents, *Bacillus subtilis* (Kono) and *B. amyloliquefaciens* FS6, on *Sclerotinia nivalis* was over 90%. After the chemical treatment, the hyphae of *Sclerotinia nivalis* YC5 were distorted and withered, and the cell membrane permeability increased, resulting in the leakage of nucleic acids and proteins; the color of the mycelia treated with biocontrol agents deepened, and balloon bubbles appeared at the tips of the hyphae. Changes such as the outflow of inclusions occurred, leading to changes in cell membrane permeability and nucleic acid and protein leakage. The ergosterol content of the strain was positively correlated with the concentration, which confirmed our results. These results lay a solid foundation for the mechanism of action of the chemical agents epoxiconazole and fludioxonil and the biocontrol agents *Bacillus amyloliquefaciens* FS6 and *Bacillus subtilis* (Kono) on *Sclerotinia nivalis* in *Panax ginseng*, and they provide strong evidence for finding a potential alternative fungicide in the prevention and control of *Sclerotinia* diseases.

## 5. Conclusions

This study identified effective pesticides for preventing and controlling ginseng *sclerotinia* root rot caused by *Sclerotinia nivalis.* The results show that the chemical agents epoxiconazole and fludioxonil and the biological control agents *B. amyloliquefaciens* FS6 and *B. subtilis* (Kono) had inhibitory effects on the mycelial growth and nucleation of *S. nivalis*. The chemical agents had a control effect of 90.60–98.16%, and the biological control agents had a control effect of 94.80–97.24%. These fungicide treatments caused malformed hyphae, reduced the ergosterol content, changed the cell membrane permeability, and increased the protein and nucleic acid permeability. Our results are of significance for guiding the prevention and treatment of ginseng *Sclerotinia* root rot disease.

## Figures and Tables

**Table 1:** Common names, contents, and manufacturers of 16 chemical fungicides.

Name	Active Ingredient Content (%)	Manufacturer
Difenoconazole	96	Hubei Jiufenglong Chemical Co., Ltd., Wuhan, China
Pyraclostrobine	96	Hebei Ruisheng Pharmaceutical Technology Co., Ltd., Shijiazhuang, China
Badistan	98	Jiangsu Rotam Chemistry Co., Ltd., Suzhou, China
Fluazinam	95	Hubei Jiufenglong Chemical Co., Ltd., Wuhan, China
Procymidone	96	Yibin Bei Chuan’an Chemical Co., Ltd., Yibin, China
Thiram	95	Hebei Guanlong Agrochemical Co., Ltd., Hengshui, China
Fluocycloprazole	97	Jiangsu Lanfeng Bio-chemical Co., Ltd., Xuzhou, China
Fluxapyroxad	98	BASF SE, Port Ludwig, Germany
Fludioxonil	95	Nantong Jiahe Chemicals Co., Ltd., Nantong, China
Thiophanate-methyl	95	Jiangsu Lanfeng Bio-chemical Co., Ltd., Xuzhou, China
Metalaxyl-M	95	Zibo Dezun Chemical Co., Ltd., Zibo, China
Myclobutanil	95	Zhejiang Heben Pesticide&Chemicals Co., Ltd., Wenzhou, China
Dimethachlon	96	Jiangxi Heyi Chemicals Co., Ltd., Jiujiang, China
Azoxystrobin	95	Zhejiang Heben Pesticide&Chemicals Co., Ltd., Wenzhou, China
Trifloxystrobin	97	Hubei Wanye Pharmaceutical Chemical Co., Ltd., Wuhan, China
Tebuconazole	98	Hailir Pesticides&Chemicals Co., Ltd., Weifang, China

**Table 2:** Common names, contents, and manufacturers of 10 biocontrol fungicides.

Biological Agent (Strain)	Content 10^8^ CFU/g (mL)	Manufacturer (Abbreviation)
*Trichoderma harzianum* T-22	3	Bioworks Co., Ltd., NY, America
*Bacillus subtilis* (Deqiang)	100	Deqiang Biotechnology Co., Ltd., Haerbin, China
*B. subtilis* (Kono)	1000	Wuhan Kono Bio-tech Co., Ltd., Wuhan, China
*Paenibacillus polymyxa*	50	Shanxi Linyi Zhongjin Chemical Co., Ltd., Yuncheng, China
*P. polymyxa* KN-03	5	Wuhan Kono Bio-tech Co., Ltd., Wuhan, China
*B. amyloliquefaciens* B7900	10	Shanxi Xiannong Biotechnology Co., Ltd.,Xian, China
*B. amyloliquefaciens* FS6	10	Laboratory of Comprehensive Management of Plant Pathology, Jilin Agricultural University (JLAU), Changchun, China.
*Bacillus velezensis* 2-8	5	Comprehensive Management of Plant Pathology, JLAU., Changchun, China.
*B. subtilis* Y4	5	Comprehensive Management of Plant Pathology, JLAU., Changchun, China.
*B. amyloliquefaciens* XH1	5	Comprehensive Management of Plant Pathology, JLAU., Changchun, China.

**Table 3:** The mass concentrations of the tested fungicides against *Sclerotinia nivalis* YC5.

Fungicides	Mass Concentration (mg·L^−1^)					
Epoxiconazole	5 × 10^−2^	1 × 10^−2^	8 × 10^−3^	5 × 10^−3^	1 × 10^−3^	5 × 10^−4^
Fludioxonil	5 × 10^−2^	1 × 10^−2^	5 × 10^−3^	1 × 10^−3^	5 × 10^−4^	1 × 10^−4^
Tebuconazole	1 × 10^0^	1 × 10^−1^	5 × 10^−2^	1 × 10^−2^	5 × 10^−3^	1 × 10^−3^
Fluazinam	1 × 10^1^	1 × 10^0^	1 × 10^−1^	1 × 10^−2^	5 × 10^−3^	1 × 10^−3^
Dimethachlon	1 × 10^0^	5 × 10^−1^	1 × 10^−1^	5 × 10^−2^	1 × 10^−2^	5 × 10^−3^
Fluxapyroxad	1 × 10^0^	1 × 10^−1^	5 × 10^−2^	1 × 10^−2^	8 × 10^−3^	5 × 10^−3^
Difenoconazole	5 × 10^0^	5 × 10^−1^	1 × 10^−1^	5 × 10^−2^	1 × 10^−2^	5 × 10^−3^
Azoxystrobin	5 × 10^0^	1 × 10^0^	5 × 10^−1^	1 × 10^−1^	5 × 10^−2^	1 × 10^−2^
Pyraclostrobine	1 × 10^0^	5 × 10^−1^	1 × 10^−1^	5 × 10^−2^	1 × 10^−2^	5 × 10^−2^
Thiram	1 × 10^1^	5 × 10^0^	1 × 10^0^	1 × 10^−1^	5 × 10^−2^	5 × 10^−2^
Myclobutanil	1 × 10^1^	1 × 10^0^	5 × 10^−1^	1 × 10^−1^	5 × 10^−2^	1 × 10^−2^
Procymidone	1 × 10^2^	1 × 10^1^	1 × 10^0^	1 × 10^−1^	5 × 10^−2^	1 × 10^−2^
Thiophanate-methyl	1 × 10^2^	1 × 10^1^	1 × 10^0^	5 × 10^−1^	1 × 10^−2^	1 × 10^−2^
Trifloxystrobin	1 × 10^2^	1 × 10^1^	1 × 10^0^	1 × 10^−1^	5 × 10^−2^	1 × 10^−2^
Badistan	5 × 10^2^	1 × 10^2^	1 × 10^1^	1 × 10^0^	1 × 10^−1^	5 × 10^−2^
Metalaxyl-M	5 × 10^2^	5 × 10^1^	1 × 10^1^	1 × 10^0^	5 × 10^−1^	1 × 10^−1^

**Table 4:** In vitro virulence of different fungicides against *Sclerotinia nivalis* YC5.

Fungicide Names	Regression Equation	Correlation/r	EC_50_/(mg·L^−1^)	EC_90_/(mg·L^−1^)	95% Confidence Interval (mg·L^−1^)
Epoxiconazole	y = 15.6571x + 0.4920	0.9445	0.0004	0.0052	0.0000~0.0020
Fluazinam	y = 11.5506x + 0.3127	0.8887	0.0008	0.0478	0.0003~0.0050
Dimethachlon	y = 9.4442x + 0.2167	0.9713	0.0012	0.4560	0.0011~0.1370
Fludioxonil	y = 11.3572x + 0.3516	0.9817	0.0140	0.5353	0.0002~1.1370
Difenoconazole	y = 11.5989x + 0.3767	0.9801	0.0246	0.7364	0.0100~0.1740
Fluxapyroxad	y = 8.7231x + 0.1757	0.9946	0.0006	0.9139	0.001~0.0340
Tebuconazole	y = 11.3066x + 0.3622	0.9806	0.0275	0.9407	0.0060~0.0860
Myclobutanil	y = 10.8126x + 0.3505	0.9841	0.0628	2.4207	0.0600~0.8300
Procymidone	y = 12.4986x + 0.5083	0.9409	0.3915	4.8580	0.0220~0.8240
Pyraclostrobine	y = 11.7435x + 0.4578	0.9563	0.4012	6.5695	0.0130~10.2480
Badistan	y = 9.2158x + 0.2725	0.9233	0.1912	20.9562	0.0010~0.9810
Azoxystrobin	y = 9.4945x + 0.3009	0.9954	0.3264	22.9565	0.0040~0.0100
Metalaxyl-M	y = 9.5073x + 0.3218	0.9675	0.8254	44.0798	0.2155~1.1092
Thiophanate-methyl	y = 8.7453x + 0.2744	0.9426	1.17900	125.2117	0.1370~2.4810
Thiram	y = 7.4638x + 0.1452	0.9816	0.0429	288.3154	0.0010~0.0620
Trifloxystrobin	y = 7.3871x + 0.2243	0.9429	23.9115	7189.6160	3.5950~63.9240

**Table 5:** Effects of different biocontrol fungicides (strains) on the mycelial growth of *S. nivalis* YC5.

Fungicide Names	Regression Equation	Correlation/ r	EC_50_/ (CFU·mL^−1^)	EC_90_/ (CFU·mL^−1^)	95% Confidence Interval (CFU·mL^−1^)
*Bacillus subtilis* (Kono)	y = 6.9784x + 0.0823	0.9767	3.6552 × 10^2^	2.0676 × 10^8^	0.0000~462.0690
*Paenibacillus polymyxa* (Linyi)	y = 6.7956x + 0.0883	0.9370	1.5000 × 10^3^	2.9025 × 10^9^	5.4348~7.0373 × 10^3^
*B. amyloliquefaciens* FS6	y = 7.2164x + 0.1179	0.9460	6.8000 × 10^3^	3.5505 × 10^8^	1.2740 × 10^0^~2.6092 × 10^4^
*B. subtilis* (Deqiang)	y = 6.8627x + 0.1064	0.9229	2.5000 × 10^4^	4.1835 × 10^9^	4.4258 × 10^3^~5.4075 × 10^4^
*B. subtilis* Y4	y = 6.9229x + 0.1467	0.9414	2.0253 × 10^6^	1.2484 × 10^10^	2.2271 × 10^6^~6.1604 × 10^9^
*B. amyloliquefaciens* XH1	y = 6.4724x + 0.1376	0.9420	2.2507 × 10^7^	2.4700 × 10^11^	4.6127 × 10^6^~1.8557 × 10^13^
*P. polymyxa* KN-03	y = 6.2071x + 0.1152	0.9734	2.8101 × 10^7^	1.8824 × 10^12^	1.6513 × 10^6^~2.5609 × 10^15^
*B. velezensis* 2-8	y = 8.0255x + 0.2888	0.9402	2.8216 × 10^7^	2.3726 × 10^9^	1.1886 × 10^6^~8.0405 × 10^10^
*B. amyloliquefaciens* B7900	y = 7.0324x + 0.2365	0.9670	1.8541 × 10^8^	4.1541 × 10^10^	8.3402 × 10^7^~4.1027 × 10^12^
*Trichoderma harzianum* T-22	y = 6.3308x + 0.1921	0.9955	9.7956 × 10^8^	7.6761 × 10^11^	1.7560 × 10^8^~9.4916 × 10^11^

**Table 6:** Effects of different chemicals on the number of *S. nivalis* YC5 sclerotia (30 d).

Fungicide Names	Inhibition Rate of the Formation of Sclerotia at Different Concentrations of Fungicides (%)						
	Concentration 1	Concentration 2	Concentration 3	Concentration 4	Concentration 5	Concentration 6	The Average Inhibition Rate
Epoxiconazole	76.39 ± 3.20	70.33 ± 6.32	49.14 ± 4.84	47.13 ± 3.34	43.69 ± 1.82	34.01 ± 1.21	53.45 ± 3.46
Fludioxonil	97.38 ± 4.55	96.97 ± 3.37	95.56 ± 4.25	95.16 ± 4.57	80.43 ± 1.85	72.96 ± 3.47	89.74 ± 3.68
Tebuconazole	85.27 ± 4.12	72.56 ± 1.53	34.41 ± 8.82	27.35 ± 4.84	20.28 ± 5.76	7.97 ± 3.10	41.31 ± 4.70
Fluazinam	92.73 ± 1.60	71.95 ± 3.65	64.28 ± 0.61	56.41 ± 3.03	12.62 ± 0.93	8.84 ± 5.55	51.14 ± 2.56
Dimethachlon	80.83 ± 2.29	74.98 ± 2.13	48.13 ± 1.94	32.39 ± 2.29	31.18 ± 2.73	15.04 ± 3.45	47.09 ± 2.47
Fluxapyroxad	96.16 ± 1.93	95.82 ± 4.25	94.85 ± 4.38	92.43 ± 3.17	83.64 ± 1.85	69.98 ± 1.65	88.81 ± 2.87
Difenoconazole	74.57 ± 3.37	64.48 ± 2.45	58.03 ± 2.13	54.59 ± 3.68	44.75 ± 5.45	33.40 ± 6.16	54.97 ± 3.87
Azoxystrobin	65.29 ± 22.20	60.45 ± 10.08	34.61 ± 1.60	25.94 ± 1.94	24.52 ± 5.04	21.70 ± 5.49	38.75 ± 7.73
Pyraclostrobine	73.97 ± 2.47	64.68 ± 2.43	58.03 ± 2.73	55.40 ± 2.44	51.41 ± 3.23	45.01 ± 3.15	58.08 ± 2.74
Thiram	98.59 ± 2.44	95.96 ± 2.45	84.46 ± 2.80	50.55 ± 7.02	46.92 ± 1.53	31.18 ± 14.04	67.94 ± 5.05
Myclobutanil	87.09 ± 1.53	86.88 ± 3.78	76.79 ± 2.86	68.92 ± 2.45	57.01 ± 4.84	22.70 ± 9.99	66.57 ± 4.24
Procymidone	92.72 ± 3.51	90.72 ± 4.58	85.67 ± 1.26	70.13 ± 6.18	65.08 ± 8.82	26.54 ± 3.34	71.81 ± 4.62
Thiophanate-methyl	83.65 ± 3.78	79.61 ± 1.26	59.64 ± 9.43	58.63 ± 0.93	40.67 ± 7.44	17.25 ± 2.86	56.58 ± 4.28
Trifloxystrobin	72.35 ± 4.58	64.28 ± 2.77	61.45 ± 5.22	58.22 ± 6.98	34.61 ± 2.19	0.00 ± 0.00	48.49 ± 3.62
Badistan	75.98 ± 8.51	68.52 ± 3.78	59.44 ± 1.21	53.18 ± 3.51	41.80 ± 4.60	0.58 ± 4.86	49.92 ± 4.41
Metalaxyl-M	85.88 ± 2.29	74.37 ± 2.29	65.90 ± 3.65	42.48 ± 4.57	21.70 ± 7.18	2.52 ± 7.59	48.81 ± 4.60

Note: The concentrations of the fungicides are shown in Table 3.

**Table 7:** The effects of different biocontrol agents (strains) on the number of *S. nivalis* YC5 sclerotia on PDA (30 d).

Biocontrol Agent (Strain) Names	Inhibition Rate of the Formation of Sclerotia at Different Concentrations of Biocontrol Agents (Strains) (%)						
	10^6^ CFU·mL^−1^	5 × 10^5^ CFU·mL^−1^	10^5^ CFU·mL^−1^	5 × 10^4^ CFU·mL^−1^	10^4^ CFU·mL^−1^	10^3^ CFU·mL^−1^	The Average Inhibition Rate
*Bacillus subtilis* (Kono)	100.00 ± 0.00	100.00 ± 0.00	100.00 ± 0.00	100.00 ± 0.00	100.00 ± 0.00	100.00 ± 0.00	100.00 ± 0.00
*B. subtilis* (Deqiang)	100.00 ± 0.00	100.00 ± 0.00	100.00 ± 0.00	100.00 ± 0.00	100.00 ± 0.00	100.00 ± 0.00	100.00 ± 0.00
*B. amyloliquefaciens* FS6	100.00 ± 0.00	100.00 ± 0.00	100.00 ± 0.00	100.00 ± 0.00	100.00 ± 0.00	100.00 ± 0.00	100.00 ± 0.00
*P. polymyxa (Linyi)*	100.00 ± 0.00	100.00 ± 0.00	100.00 ± 0.00	100.00 ± 0.00	100.00 ± 0.00	100.00 ± 0.00	100.00 ± 0.00
*P. polymyxa* KN-03	100.00 ± 0.00	100.00 ± 0.00	100.00 ± 0.00	100.00 ± 0.00	94.17 ± 2.35	81.21 ± 3.61	95.90 ± 0.99
*B. subtilis* Y4	100.00 ± 0.00	98.90 ± 0.17	96.70 ± 1.83	96.33 ± 4.07	96.33 ± 4.13	94.91 ± 3.20	97.20 ± 2.23
*B. amyloliquefaciens* XH1	73.47 ± 4.84	70.09 ± 2.66	64.59 ± 2.58	49.98 ± 3.46	44.59 ± 7.52	33.63 ± 4.73	56.06 ± 4.30
*Bacillus velezensis* 2-8	98.35 ± 2.67	88.26 ± 4.13	81.47 ± 8.57	78.16 ± 2.11	69.36 ± 6.35	35.76 ± 2.14	75.23 ± 4.33
*Trichoderma harzianum* T-22	100.00 ± 0.00	100.00 ± 0.00	100.00 ± 0.00	96.02 ± 2.52	70.64 ± 2.12	46.87 ± 6.30	85.59 ± 1.82
*B. amyloliquefaciens* B7900	98.66 ± 2.29	94.86 ± 1.90	92.66 ± 4.22	90.46 ± 4.24	84.59 ± 6.73	75.15 ± 11.65	89.40 ± 5.17

**Table 8:** Field control effects of the different agents on *Sclerotinia nivalis* YC5 (Changchun, 2022, 2023).

Agents	Concentration (mg·L^−1^ or CFU·mL^−1^)	Control Effect (%)	
		2022	2023
Epoxiconazole	0.052	98.16 ± 1.14	90.60 ± 2.61
Fludioxonil	5.353	98.16 ± 0.79	94.87 ± 3.49
*Bacillus subtilis* (Kono)	2.0676 × 10^8^	97.04 ± 1.13	94.80 ± 2.32
*B. amyloliquefaciens* FS6	3.5505 × 10^8^	97.24 ± 3.13	95.73 ± 1.26
CK	--	--	--

**Table 9:** Comparison of the ergosterol content after the different treatments.

Agents	Concentration (mg·L^−1^ or CFU·mL^−1^)	Ergosterol Content (μg·mL^−1^)
Epoxiconazole	0.0520	8.63 ± 0.13 h
	0.0005	4.20 ± 0.04 i
Fludioxonil	5.3530	104.13 ± 2.27 b
	0.0535	30.68 ± 0.95 g
*Bacillus subtilis* (Kono)	2.0676 × 10^9^	85.53 ± 1.32 c
	2.0676 × 10^7^	37.66 ± 0.35 f
*B. amyloliquefaciens* FS6	3.5505 × 10^9^	77.05 ± 1.42 d
	3.5505 × 10^7^	41.38 ± 0.24 e
CK	--	197.87 ± 3.21 a

Note: The letters a–i represent different levels of differences between treatments. The treatment with the highest average effectiveness over different time periods is labeled as “a”. Then, each average is compared with this reference average. If the difference is not significant, it is also labeled as “a”. This process continues until one average is significantly different, which is labeled as “b”. The rest of the averages are labeled accordingly in a similar manner.

## Data Availability

The original contributions presented in the study are included in the article, further inquiries can be directed to the corresponding authors.
